# Patterns of Insertion and Deletion in Mammalian Genomes

**DOI:** 10.2174/138920207783406479

**Published:** 2007-09

**Authors:** Yanhui Fan, Wenjuan Wang, Guoji Ma, Lijing Liang, Qi Shi, Shiheng Tao

**Affiliations:** Bioinformatics Center, College of Life Science, Northwest A&F University, Yangling, Shaanxi 712100, China

**Keywords:** Insertion, deletion, gap, indel, mammalian genome.

## Abstract

Nucleotide insertions and deletions (indels) are responsible for gaps in the sequence alignments. Indel is one of the major sources of evolutionary change at the molecular level. We have examined the patterns of insertions and deletions in the 19 mammalian genomes, and found that deletion events are more common than insertions in the mammalian genomes. Both the number of insertions and deletions decrease rapidly when the gap length increases and single nucleotide indel is the most frequent in all indel events. The frequencies of both insertions and deletions can be described well by power law.

## INTRODUCTION

With the successful completion of the genome sequencing projects, the challenge is now to understand the instructions encoded in the genomes. The comparative genomic analysis by cross-species alignment of mammalian genomes is one of the most powerful ways to decipher the evolutionary process of mammalian genomes. One major aim of genomics research is to identify differences between genomes of species or individuals. The differences of genomes require genetic variation. One mechanism that increases genetic variation is mutation. There are many kinds of mutations. A mutation in which one “letter” of the genetic code is changed to another is a point mutation. Lengths of DNA be deleted or inserted in a gene means a deletion or insertion, respectively. Finally, genes or parts of genes can become inverted or duplicated. Previous researches unveiled that insertions and deletions, instead of substitutions, comprise the majority of the genomic divergence [1-4]. Therefore, the study of the patterns of insertion and deletion is necessary to understand the mammalian evolution. 

By examining the homologous protein sequences, de Jong and Rydén (1981) observed that deletions of amino acids occurred about four times more frequently than insertions [5]. Deletion events also outnumbered insertions for processed pseudogenes [6-9]. Deletions are about twice as frequent as insertions for nuclear DNA, and in mitochondrial DNA, deletions occur at a slightly higher frequency than insertions [10]. Deletion events are also found more common than insertions in both mouse and rat [11-13].

There were several studies that focused on the size distribution of insertions and deletions. The exhaustive matching of the protein sequence database found that a power law with an exponent of 1.7 approximates quite closely the observed gap (insertion and deletion) length distribution [14]. The studies of pseudogenes suggested that the size distribution of insertions and deletions can be empirically described by power law [7,  9]. Qian and Goldstein (2001) examined gaps occured in FSSP database [15], using alignments based on their common structures, and they fitted the probability distribution of gap length to a quadruple exponential function [16]. Goonesekere and Lee (2004) examined the pattern of gaps of 3992 structurally aligned protein domain pairs in SCOP database [17], they found that the distributions of the logarithm of the probability of gaps varies linearly with the length of gap with a break at the gap of length 3 [18]. 

In this research, the multiple alignments of 19 mammalian genomes were used to analyze the patterns of insertions and deletions. We tested whether deletions always occur more frequently than insertions. Then we studied the length distributions of insertions and deletions.

## MATERIALS AND METHODS

The multiple alignments of 28 vertebrate species were downloaded from UCSC Genome Bioinformatics website [19]. Table **1** shows the genome assemblies that were included in the 28-way multiple alignments. Table **2** shows the data used in this research.

The 28-way multiple alignments were built as follows. Firstly, lineage-specific repeats were removed prior to alignment, then pairwise alignments with the human genome were generated for each species using BLASTZ [20] from repeat-masked genomic sequence. Pairwise alignments were then linked into chains using AXTCHAIN [21] that finds maximally scoring chains of gapless subsections of the alignments organized in a k-dimensional tree. Then CHAINNET [21] was used to produce an alignment net. The resulting best-in-genome pairwise alignments were progressively aligned using MULTIZ [22], based on the phylogenetic tree [23], as Fig. (**1**) shows, to produce multiple alignments.

**Fig. (2):**
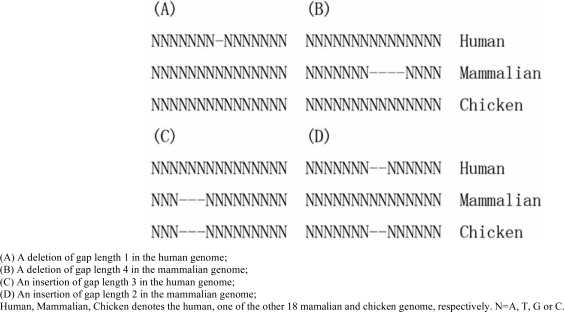
Definition of insertions and deletions.

Only the multiple alignments of 19 mammalian species were studied. The triple alignments of human, chicken and one of the other 18 mammalian species were used to assign the insertions and deletions to human or the other mammalian species by the parsimony principle, using chicken as outgroup. In this study, there were four events inferred as insertions or deletions (Fig. **2**).

The probability of an insertion or deletion of length k was calculated by equation 1 where f_k_ is the probability of the insertion or deletion with the gap length k, N_k_ is the number of the insertion or deletion that has the gap length k. Then the power law can be defined as equation 2 [9].

(1) $$f_{k}=\frac{N_{k}}{\sum_{k=1}^{\infty }N_{K}}$$

(2) $$f_{k}=a^{*}k^{-b}$$

## RESULTS

Fig. (**3**) shows the length distributions of the insertions and deletions of the 18 mammalian genomes. Deletions occur more frequently than insertions over all gap lengths. However, in opossum, insertions occur more frequently than deletions except the gap of length 2. The ratio of deletions to insertions varies from 0.85 to 12.82 (Table **3**). Only in the opossum the ratio is less than 1. In rabbit, the deletions are extremely more than insertions. The total lengths of deletions are larger than insertions, except for hedgehog, elephant, tenrec and opossum.

Both the number of insertions and deletions decrease rapidly with the increases of gap length. The single nucleotide insertion and deletion are the most frequent in all events. The percentage of single nucleotide insertions varies from 28.63% to 71.00%, and the percentage of single nucleotide deletions varies from 26.54% to 46.74% (Table **3**).

The probability of insertions and deletions, as a function of gap length, fits power law equation given above very well. Regression analysis of the data, using SPSS 15.0 [24], gave the values of a, b and R^2^ (Table **4**). SPSS was also used to perform the Kolmogorov-Smirnov test for goodness-of-fit tailored to power law distributions. Table **4** shows the results of the test. Fig. (**4**) shows the plots of parameters k and f_k _for deletions. Fig. (**5**) shows the plots of k and f_k _for insertions.

## DISCUSSION

Nucleotide substitution, insertion and deletion (indel) events are the major driving forces that have shaped genomes [9]. Furthermore, recent researches found that insertions and deletions, instead of substitutions, are the major path to the genomic divergence [1-4]. Therefore, the study of the patterns of insertion and deletion in the genomes is essentially important.

Previous studies found that there was preponderance of deletions over insertions [5-13]. From the extensive genome data used in this study, we have shown that deletions occur more frequently than insertions in genomes. Although insertions are more frequent than deletions in opossum, it is not significant. Therefore, deletions occur more frequently than insertions can be regarded as a general genomic feature.

Single nucleotide insertion and deletion are the most frequent in all events, and the frequency of insertions and deletions decrease quickly as the gap length increases. The high occurrence of single nucleotide gaps was also observed in the study of 22 human and 30 rodents processed pseudogenes [6], 78 human processed pseudogenes [7], 1726 human ribosomal protein pseudogene sequences [9], noncoding nucleotide sequences of primates [10], Escherichia coli [25], chloroplast noncoding nucleotide sequence of nine monocot plants [26]. Therefore, the high percent of single nucleotide insertion and deletion seems to be a common phenomenon in the genomic evolution.

Benner *et al*. (1993) studied the alignments of homologous protein sequence pairs and concluded that the distribution of the gap length follows power law distribution [14]. Gu and Li (1995) aligned 78 human processed pseudogenes, the human functional genes and the reference, they found the size distributions of insertions and deletions fitted to power law very well [7]. Recently, Zhang and Gerstein (2003) examined the patterns of insertions and deletions in 1726 processed ribosomal protein pseudogenes and found that the frequencies of both insertions and deletions followed characteristic power law behavior associated with the length of the gaps [9]. In this study, the probability distributions of insertions and deletions in the 18 mammalian genomes can both be described by power law distribution. The results suggest that the gap penalty should be log-affine [27], i.e., g(k)=a+bk+clnk, where g(k) is the gap penalty for insertion or deletion, k is the length of the insertion or deletion.

## Figures and Tables

**Fig. (1):**
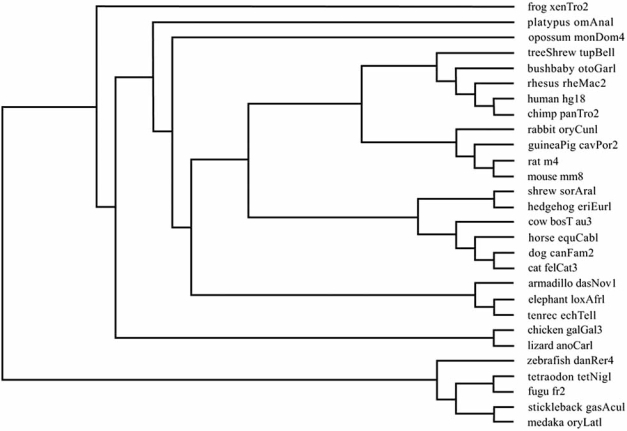
Phylogenetic tree of 28 vertebrate species.

**Fig. (3):**
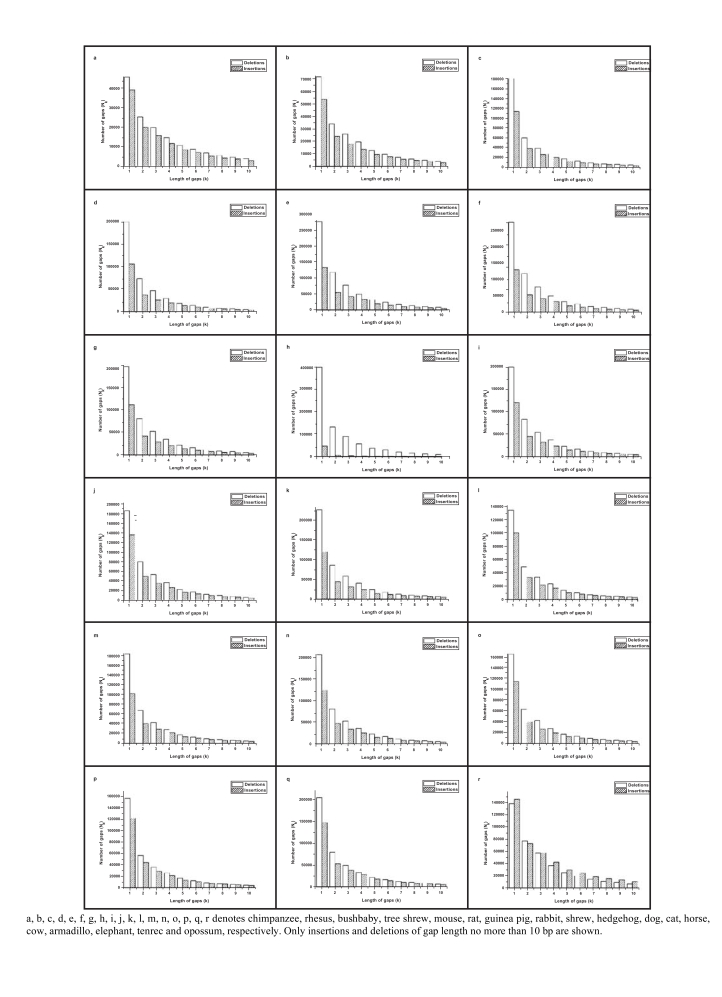
Length distributions of insertions and deletions.

**Fig. (4):**
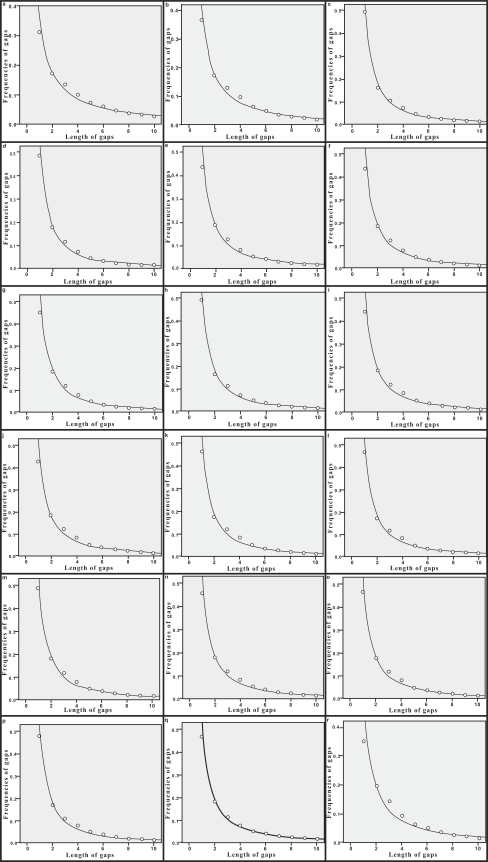
f_**k**_ vs k plotting for deletions.

**Fig. (5):**
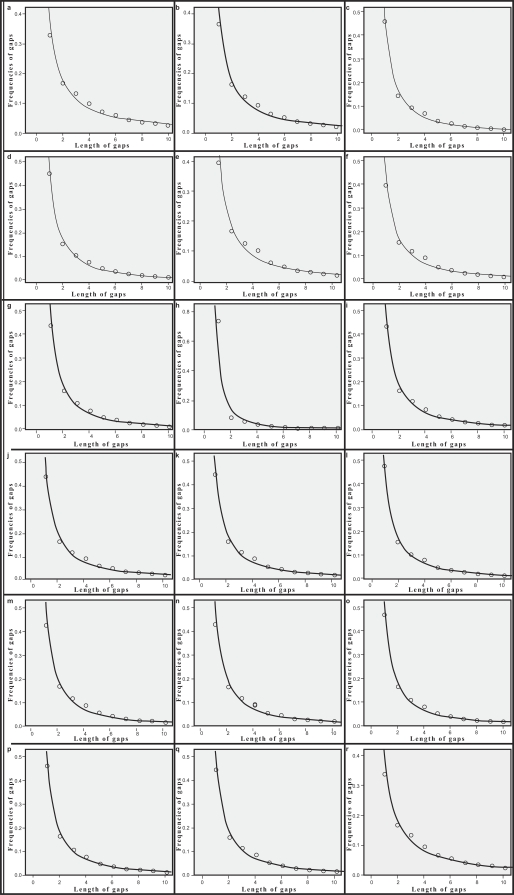
f_**k**_ vs k plotting for insertions.

**Table 1:** Genome Assemblies Included in the 28-way Multiple Alignments

Organism	Species	Release Date	UCSC Version
human	Homo sapiens	Mar 2006	hg18
chimpanzee	Pan troglodytes	Mar 2006	panTro2
rhesus	Macaca mulatta	Jan 2006	rheMac2
bushbaby	Otolemur garnetti	Dec 2006	otoGar1
tree shrew	Tupaia belangeri	Dec 2006	tupBel1
mouse	Mus musculus	Feb 2006	mm8
rat	Rattus norvegicus	Nov 2004	rn4
guinea pig	Cavia porcellus	Oct 2005	cavPor2
rabbit	Oryctolagus cuniculus	May 2005	oryCun1
shrew	Sorex araneus	June 2006	sorAra1
hedgehog	Erinaceus europaeus	June 2006	eriEur1
dog	Canis familiaris	May 2005	canFam2
cat	Felis catus	Mar 2006	felCat3
horse	Equus caballus	Feb 2007	equCab1
cow	Bos taurus	Aug 2006	bosTau3
armadillo	Dasypus novemcinctus	May 2005	dasNov1
elephant	Loxodonta africana	May 2005	loxAfr1
tenrec	Echinops telfairi	July 2005	echTel1
opossum	Monodelphis domestica	Jan 2006	monDom4
platypus	Ornithorhychus anatinus	Mar 2007	ornAna1
lizard	Anolis carolinensis	Feb 2007	anoCar1
chicken	Gallus gallus	May 2006	galGal3
frog	Xenopus tropicalis	Aug 2005	xenTro2
fugu	Takifugu rubripes	Oct 2004	fr2
tetraodon	Tetraodon nigroviridis	Feb 2004	tetNig1
stickleback	Gasterosteus aculeatus	Feb 2006	gasAcu1
medaka	Oryzias latipes	Apr 2006	oryLat1
zebrafish	Danio rerio	Mar 2006	danRer4

**Table 2:** The Details about the Data

Name	Last Modified	Size	Name	Last Modified	Size
chr1.maf.gz	2007-5-30 17:09	822M	chr13.maf.gz	2007-5-30 17:27	338M
chr2.maf.gz	2007-5-30 17:52	878M	chr14.maf.gz	2007-5-30 17:31	321M
chr3.maf.gz	2007-5-30 18:04	735M	chr15.maf.gz	2007-5-30 17:34	289M
chr4.maf.gz	2007-5-30 18:10	636M	chr16.maf.gz	2007-5-30 17:37	278M
chr5.maf.gz	2007-5-30 18:16	641M	chr17.maf.gz	2007-5-30 17:40	284M
chr6.maf.gz	2007-5-30 18:22	609M	chr18.maf.gz	2007-5-30 17:42	271M
chr7.maf.gz	2007-5-30 18:28	528M	chr19.maf.gz	2007-5-30 17:44	140M
chr8.maf.gz	2007-5-30 18:33	499M	chr20.maf.gz	2007-5-30 17:54	219M
chr9.maf.gz	2007-5-30 18:37	418M	chr21.maf.gz	2007-5-30 17:56	110M
chr10.maf.gz	2007-5-30 17:14	475M	chr22.maf.gz	2007-5-30 17:57	107M
chr11.maf.gz	2007-5-30 17:19	474M	chrX.maf.gz	2007-5-30 18:41	405M
chr12.maf.gz	2007-5-30 17:24	460M	chrY.maf.gz	2007-5-30 18:41	23M

**Table 3:** Ratios of Deletions to Insertions and the Percentage of the Single Nucleotide Gap

Organism	RN	RL	P	
			Insertion	Deletion
chimpanzee	1.26 : 1	1.32 : 1	28.63%	26.54%
Rhesus	1.34 : 1	1.20 : 1	32.52%	32.50%
Bushbaby	1.48 : 1	1.10 : 1	42.44%	46.13%
tree shrew	1.66 : 1	1.03 : 1	40.53%	45.93%
Mouse	1.78 : 1	1.24 : 1	35.31%	40.87%
Rat	1.87 : 1	1.32 : 1	36.14%	41.17%
guinea pig	1.69 : 1	1.26 : 1	40.50%	42.21%
Rabbit	12.82 : 1	17.26 : 1	71.00%	46.74%
Shrew	1.54 : 1	1.04 : 1	38.82%	41.77%
Hedgehog	1.34 : 1	0.96 : 1	39.44%	40.20%
Dog	1.72 : 1	1.26 : 1	39.80%	43.72%
Cat	1.34 : 1	1.11 : 1	43.33%	43.19%
Horse	1.54 : 1	1.18 : 1	38.89%	45.64%
Cow	1.49 : 1	1.03 : 1	38.38%	42.67%
Armadillo	1.43 : 1	1.20 : 1	42.88%	43.35%
Elephant	1.21 : 1	0.93 : 1	41.78%	44.28%
Tenrec	1.29 : 1	0.92 : 1	39.88%	43.03%
Opossum	0.85 : 1	0.60 : 1	29.27%	32.74%

RN denotes the ratio between the total number of deletions and insertions. RL denotes the ratio between the total length of deletions and insertions. P denotes the percentage of the single nucleotide insertion or deletion.

**Table 4:** Estimates of the Parameters

Organism	a		b		R^2^		D*		P-value**	
	Del	Ins	Del	Ins	Del	Ins	Del	Ins	Del	Ins
chimpanzee	0.369	0.381	1.059	1.096	0.975	0.976	0.100	0.100	1.0	1.0
Rhesus	0.442	0.414	1.259	1.193	0.974	0.984	0.100	0.100	1.0	1.0
Bushbaby	0.528	0.492	1.537	1.434	0.993	0.989	0.100	0.100	1.0	1.0
tree shrew	0.572	0.481	1.624	1.400	0.990	0.989	0.100	0.100	1.0	1.0
Mouse	0.528	0.464	1.492	1.324	0.986	0.974	0.100	0.100	1.0	1.0
Rat	0.532	0.470	1.502	1.342	0.986	0.975	0.100	0.100	1.0	1.0
guinea pig	0.546	0.500	1.538	1.433	0.985	0.986	0.100	0.100	1.0	1.0
Rabbit	0.547	0.510	1.573	1.883	0.989	0.977	0.100	0.100	1.0	1.0
Shrew	0.550	0.474	1.539	1.372	0.981	0.989	0.100	0.100	1.0	1.0
Hedgehog	0.529	0.474	1.487	1.376	0.981	0.988	0.100	0.100	1.0	1.0
Dog	0.557	0.476	1.569	1.384	0.983	0.989	0.100	0.100	1.0	1.0
Cat	0.534	0.487	1.526	1.436	0.987	0.992	0.100	0.100	1.0	1.0
Horse	0.557	0.486	1.591	1.393	0.992	0.984	0.100	0.100	1.0	1.0
Cow	0.542	0.475	1.530	1.369	0.985	0.986	0.100	0.100	1.0	1.0
Armadillo	0.539	0.502	1.536	1.457	0.989	0.990	0.100	0.100	1.0	1.0
Elephant	0.532	0.491	1.527	1.433	0.990	0.994	0.100	0.100	1.0	1.0
Tenrec	0.515	0.482	1.487	1.398	0.994	0.988	0.100	0.100	1.0	1.0
Opossum	0.473	0.391	1.325	1.123	0.962	0.979	0.100	0.100	1.0	1.0

* The maximum difference between the cumulative distributions in the KS-test.

** The P-value of the KS-test.

All data analyses were performed using SPSS version 15.0 (SPSS 2006).
